# Supplementary material for: Sleep Duration, Midday Napping, and Serum Homocysteine Levels: A Gene–Environment Interaction Study
Source: Nutrients. 2023 Jan 1;15(1):210. doi: 10.3390/nu15010210 (PMC9823917; doi:10.3390/nu15010210)
Supplement: Supplementary file 1 [file nutrients-15-00210-s001.zip › nutrients-2078402-supplementary.pdf]

Supplemental Material

Sleep duration, midday napping, and serum homocysteine levels: a gene-environment interaction study

Tingting Mo [1], Yufei Wang [1], Hui Gao [1], Wending Li [1], Lue Zhou [1], Yu Yuan [1], Xiaomin Zhang [1], Meian He [1], Huan Guo [1], Pinpin Long [1,*], Tangchun Wu [1]

[1] Department of Occupational and Environmental Health, Key Laboratory of Environment and Health, Ministry of Education and State Key Laboratory of Environmental Health (Incubating), School of Public Health, Tongji Medical College, Huazhong University of Science and Technology, Wuhan, China

[*] Correspondence: longpinpintjmu@163.com; Tel. : +86-27-83692347

Supplementary Methods. Definitions of covariates.

Supplementary Table S1. Baseline characteristics of the total participants and participants with genetic data.

Supplementary Table S2. Association of sleep duration and midday napping with serum homocysteine levels with additional adjustment for occupational factors.

Supplementary Table S3. Association of 24-hour sleep duration with serum homocysteine levels.

Supplementary Table S4. Information on 18 Hcy-related SNPs in the present study and the reported meta-analysis.

Supplementary Table S5. The estimated difference in ln-transformed homocysteine levels associated with GRS.

Supplementary Table S6. The estimated difference in ln-transformed homocysteine levels associated with GRS-6.

Supplementary Figure S1. Association of sleep duration and midday napping with serum homocysteine levels stratified by baseline characteristics.

Supplementary Figure S2. Serum homocysteine (Hcy) levels according to combined categories of sleep duration and midday napping.

Supplementary Figure S3. Serum homocysteine (Hcy) levels according to combined categories of GRS-6 with sleep duration and midday napping.

Supplementary Figure S4. The schematic overview of two major pathways of homocysteine metabolism.

Definitions of covariates

Body mass index was calculated as weight in kilograms divided by height in meters squared. The estimated glomerular filtration rate was calculated using the Chronic Kidney Disease Epidemiology Collaboration equation based on creatinine levels, age, sex, and ethnicity.[1] Education level included primary school or below, junior high school, senior high school, and college or above. Regular exercise was defined as exercised (walking, jogging, cycling, dancing, doing tai chi, playing ball games, swimming, or any kinds of exercises) ≥5 times/week, 30 minutes each time and lasted for at least six months. Dietary consumption on meats, milk or dairy products, beans or soy products, fishes or seafoods, and fruits or vegetables were recorded, and participants were further categorised into two groups according to their frequency of consumption (≥5 times/week; yes, no). Hypertension was defined as blood pressure ≥140/90 mmHg, or with self-reported history of hypertension, or use of anti-hypertensive medications. Dyslipidemia was defined as total cholesterol ≥6.22 mmol/L, or triglyceride ≥2.26 mmol/L, or high-density lipoprotein cholesterol <1.04 mmol/L, or low-density lipoprot4.14 mmol/L, or with self-reported history of dyslipidemia, or use of lipid-lowing medications. Participants were identified as having diabetes, if they had fasting glucose ≥7.0 mmol/L, or self-reported history of diabetes, or used anti-diabetes medications. Individuals who were smoking at least one cigarette per day in the past six months were identified as current smokers; and those who were drinking alcohol (wine, liquor, or beer) at least one time per week in the past six months were considered as current drinkers. Sleep quality was assessed by the question "How was your sleep quality at night in the past six months?" with 4 possible answers: "Good" "Fair" "Poor" or "very poor, and had to use hypnotics frequently" and participants responded "very poor" were merged into the poor group. In the DFTJ cohort, we defined the past shift

work was defined as experience of any irregular work schedule, as opposed to the normal daytime working hours [1]. Information on past shift work was collected acquired using by self-administrated questionnaire through face-to-face interview. Participants Subjects were asked, “Have you ever participated in shift work during your past occupational life?” Those who answered “Yes”, were asked for their total years of past shift work were asked. The duration of past shift work was rounded to 0.25 years, and was treated in both 5-year intervals and categorized as none, ≤5.00, 5.25-10.00, 10.50-20.00, and >20.00 years according to the distribution of our study population and our previous study [2].

Consistently in the Dongfeng-Tongji cohort [2], job titles were obtained through self-administrated questionnaire and were categorized into three categories: manufacturing or manual labor work, services or sales work and office work. For those who had switched their jobs, the most deleterious one that lasted >5 years was presented.

Supplementary Table S1. Baseline characteristics of participants with genetic data according to sleep duration and midday napping (N=15,126).

Variables	Sleep duration, hours				Midday napping, minutes				
	<7	7 to <8	8 to <9	≥9	0	1-30	31-60	61-90	>90
Sample size, n	885	3691	6423	4127	6928	2188	3855	1272	883
Age, years	63.4 (7.9)	62.7 (7.6)	63.2 (7.9)	63.5 (8.6)	62.2 (7.8)	63.1 (8.0)	64.3 (8.1)	64.8 (8.1)	64.1 (8.1)
Female, (%)	57.5	57.4	57.7	54.8	61.5	63.0	50.4	42.4	46.5
Male, (%)	42.5	42.6	42.3	45.2	38.5	37	49.6	57.6	53.5
Education levels, (%)									
Primary school or below	17.1	17.5	20.2	25.7	19.8	21.8	19.5	18.1	25.0
Middle school	36.3	35.9	38.6	38.5	34.3	39.1	37.3	37.7	39.6
High school or beyond	46.1	45.9	40.7	35.2	45.2	38.5	42.7	43.9	35.3
BMI, kg/m^2	24.3 (3.1)	24.1 (3.1)	23.9 (3.1)	23.8 (3.2)	23.9 (3.1)	23.9 (3.1)	24.0 (3.1)	24.1 (3.1)	24.1 (3.1)
eGFR, mL/min/1.73 m^2	83.1 (16.1)	83.8 (15.4)	82.9 (15.9)	81.6 (17.0)	83.1 (16.0)	83.1 (16.3)	82.3 (15.6)	81.3 (17.1)	83.3 (16.4)
Hypertension, (%)	59.6	58.3	59.4	60.2	59.6	56.0	63.0	65.2	65.7
Dyslipidemia, (%)	39.6	40.7	38.7	38.4	38.7	37.1	39.4	42.2	47.5

Diabetes, (%)	18.7	19.8	19.0	20.7	20.1	17.3	21.3	22.2	22.4
Smoking status, (%)									
Current smoker	16.4	20.1	15.6	17.8	13.7	15.8	17.3	20.3	22.8
Former smoker	10.3	10.6	10.1	11.3	9.0	8.3	12.6	15.9	14.9
Never smoker	73.3	69.3	74.3	70.9	77.2	75.9	70.2	63.8	62.3
Alcohol intake status, (%)									
Current drinker	27.0	28.0	25.0	25.9	23.5	23.4	28.1	29.7	35.8
Former drinker	4.8	4.7	4.8	5.2	4.9	4.0	4.8	9.4	6.5
Never drinker	68.2	67.2	70.2	68.9	71.6	72.6	67.1	60.8	57.8
Dietary intakes [a], (%)									
Meats	53.8	53.7	53.3	49.6	55.0	51.9	52.7	50.6	51.9
Milk or dairy products	43.1	46.2	42.5	39.2	44.5	40.3	44.5	41.0	39.1
Beans or soy products	56.6	57.1	56.0	52.7	57.4	53.6	57.5	54.3	55.6
Fishes or seafoods	22.2	21.5	22.4	21.1	23.2	22.4	22.2	18.5	19.8
Fruits or vegetables	97.0	97.2	96.4	96.7	96.8	96.8	96.8	95.8	95.9
Regular exercise [b], (%)	72.1	68.4	69.8	63.6	72.2	65.9	71.2	71.0	65.7

Snoring, (%)	43.6	42.4	40.3	40.0	44.6	34.9	45.7	49.4	49.2
Sleep quality, (%)									
Good	35.6	31.0	37.4	38.7	34.7	38.2	35.8	34.8	40.5
Fair	49.7	44.1	50.5	48.6	50.8	46.5	52.3	55.3	47.5
Poor	14.7	25.0	12.1	12.8	14.5	15.3	11.9	9.9	12
Job category, (%)									
Manufacturing or manual labor	44.8	45.5	45.3	47.9	44.2	48.1	43.4	42.9	47.5
Service or sales work	30.0	27.8	29.6	27.8	30.5	28.1	30.2	29.8	28.4
Office work	13.6	15.0	12.3	11.1	13.4	11.5	13.3	15.1	10.2
Past shift work, years									
None	45.7	42.9	44.4	43.0	44.3	44.2	44.7	45.6	39.8
≤5.00	13.7	15.5	13.6	13.9	14.4	13.6	14.1	13.4	14.0
5.25-10.00	10.7	9.7	10.6	11.9	10.7	11.5	10.3	10.4	11.4
10.50-20.00	11.9	12.1	10.9	11.2	11.4	11.7	11.1	9.2	12.0
>20.00	7.3	9.3	8.3	7.5	8.2	7.6	7.4	9.7	8.9
Genetic risk score									
GRS	15.5 (2.6)	15.7 (2.6)	15.7 (2.6)	15.6 (2.6)	15.7 (2.6)	15.6 (2.6)	15.7 (2.6)	15.6 (2.7)	15.7 (2.7)
GRS-6	6.1 (2.0)	6.2 (2.1)	6.2 (2.1)	6.2 (2.1)	6.2 (2.1)	6.2 (2.1)	6.2 (2.1)	6.2 (2.1)	6.2 (2.1)

BMI = body mass index; eGFR = estimated glomerular filtration rate; GRS = genetic risk score (based on 18 Hcy-related genetic variants obtained from the reported meta-analysis); GRS-6 = genetic risk score based on 6 Hcy-related genetic variants that were verified in the present study (p <0.05/18).

Characteristics are presented as mean (standard deviation) for continuous variables and percentages for categorical variables.

[a] Consumption frequency ≥5 times/week

[b] Regular exercise was defined as exercised ≥30 minutes ≥5 times/week and lasted for at least six months.

Supplementary Table S2. Association of sleep duration and midday napping with serum homocysteine levels with additional adjustment for occupational factors.

	Homocysteine [a], β (95% confidence interval)	
	Model [b]	*p* value
Sleep duration, hours		
<7	0.004 (-0.022, 0.03)	0.74
7 to <8	0.000 (ref)	
8 to <9	0.012 (-0.002, 0.027)	0.10
≥9	**0.045 (0.029, 0.061)**	**<0.0001**
Midday napping, minutes		
0	0.013 (-0.004, 0.030)	0.14
1-30	0.003 (-0.015, 0.022)	0.73
31-60	0.000 (ref)	
61-90	-0.009 (-0.033, 0.016)	0.50
>90	**0.028 (0.001, 0.056)**	**<0.05**

CI=confidence interval; Hcy=homocysteine.

[a] Homocysteine levels were natural logarithmic transformed to approximate normal distribution.

[b] The model was adjusted for age (continuous), sex (female, male), education levels (primary school or below, middle school, high school or beyond), body mass index (continuous), estimated glomerular filtration rate (continuous), hypertension (yes, no), dyslipidemia (yes, no), diabetes (yes, no), smoking status (current, ever, never), drinking status (current, ever, never), dietary intakes of meats, milk or dairy products, beans or soy products, fishes or seafoods, and fruits or vegetables (≥5 times/week; yes, no), regular exercise (yes, no), snoring (yes, no), sleep quality (good, fair, poor), job category (manufacturing or manual labor work, services or sales work, office work), and duration of past shift work (none, ≤5.00, 5.25-10.00, 10.50-20.00, and >20.00 years).

Supplementary Table S3. Association of 24-hour sleep duration with serum homocysteine levels.

	Homocysteine [a], β (95% confidence interval)		
	Model 1 [b]	Model 2 [c]	Model 3 [d]
24-hour sleep duration, hours			
<8	0.017 (-0.002, 0.036)	0.009 (-0.009, 0.026)	0.018 (-0.001, 0.037)
8 to <9	0.000 (ref)	0.000 (ref)	0.000 (ref)
9 to <10	0.024 (-0.013, 0.061)	0.015 (-0.020, 0.049)	0.018 (-0.019, 0.054)
≥10	**0.048 (0.030, 0.067)**	**0.030 (0.013, 0.046)**	**0.042 (0.023, 0.062)**

[a] Homocysteine levels were natural logarithmic transformed to approximate normal distribution.

[b] Model 1 adjusted for age (continuous) and sex (female, male).

[c] Model 2 additionally adjusted for education levels (primary school or below, middle school, high school or beyond), body mass index (continuous), estimated glomerular filtration rate (continuous), hypertension (yes, no), dyslipidemia (yes, no), diabetes (yes, no), smoking status (current, ever, never), drinking status (current, ever, never), dietary intakes of meats, milk or dairy products, beans or soy products, fishes or seafoods, and fruits or vegetables (≥5 times/week; yes, no), regular exercise (yes, no), snoring (yes, no) and sleep quality (good, fair, poor).

[d] Model 3 additionally adjusted for job category (manufacturing or manual labor work, services or sales work, office work) and duration of past shift work (none, ≤5.00, 5.25-10.00, 10.50-20.00, and >20.00 years)

Supplementary Table S4. Information on 18 homocysteine-related SNPs in the present study and the reported meta-analysis.

				Present study (N = 15,126)				Reported meta-analysis (N = 44,147)[a]			
SNP	Chr	Position	Nearest gene	Effect allele	EAF	β	*p*	Effect allele	MAF	β	*p*
**rs12134663**	**1**	**11778589**	***MTHFR***	**A**	**0.90**	**0.0401**	**1.07E-06**	A	0.80	0.1010	2.54E-21

**rs1801133**	**1**	**11796321**	***MTHFR***	**T**	**0.54**	**0.1128**	**2.13E-113**	T	0.34	0.1583	4.34E-104
rs4660306	1	45513003	*MMACHC*	T	0.09	0.0181	3.90E-02	T	0.33	0.0435	2.33E-09
rs2275565	1	236885376	*MTR*	G	0.84	0.011	1.09E-01	G	0.79	0.0542	1.96E-10
**rs1047891**	**2**	**210675783**	***CPS1***	**A**	**0.16**	**0.0422**	**6.20E-10**	A	0.33	0.0864	2.17E-10
rs548987	6	25869143	*SLC17A3*	C	0.13	0.0062	4.30E-01	C	0.13	0.0597	4.58E-27
**rs9369898**	**6**	**49414480**	***MUT***	**A**	**0.43**	**0.0193**	**1.11E-04**	A	0.62	0.0449	1.97E-08
rs42648	7	90348446	*GTPBP10*	G	0.24	0.0069	2.32E-01	G	0.40	0.0395	1.12E-08
rs1801222	10	17114152	*CUBN*	A	0.18	0.0088	1.74E-01	A	0.34	0.0453	8.43E-10

rs12780845	10	17181245	*CUBN*	A	0.77	0.0112	5.65E-02	A	0.65	0.0529	7.80E-10
**rs7130284**	**11**	**89415204**	***NOX4***	**C**	**0.82**	**0.0411**	**1.98E-10**	C	0.93	0.1242	1.88E-20
rs957140	11	89468459	*NOX4*	G	0.47	0.0026	6.02E-01	G	0.45	0.0450	2.43E-08
rs2251468	12	120967323	*HNF1A*	C	0.43	0.0109	2.99E-02	C	0.35	0.0512	1.28E-12
rs154657	16	89641688	*DPEP1*	A	0.02	-0.0088	5.88E-01	A	0.47	0.0963	1.74E-43
rs12921383	16	89793345	*DPEP1/FANCA*	C	0.10	0.0157	5.76E-02	C	0.13	0.0900	8.22E-11
rs838133	19	48756272	*FUT2*	A	0.01	-0.0222	7.49E-03	A	0.45	0.0422	7.48E-09
rs234709	21	43066854	*CBS*	C	0.90	0.0051	8.57E-01	C	0.55	0.0718	3.90E-24

**rs2851391**	**21**	**43067294**	***CBS***	**T**	**0.27**	**0.0175**	**1.68E-03**	T	0.47	0.0560	1.70E-12

Chr = chromosome; EAF = effect allele frequency; SNP = single nucleotide polymorphism.

The effect coefficients (β) and *p* values were calculation by generalized linear regression models with adjustments for age, sex and the first ten principal components.

[a] van Meurs JBJ, et al. Common genetic loci influencing plasma homocysteine concentrations and their effect on risk of coronary artery disease. *Am J Clin Nutr*. 2013; **98** (3): 668-676. doi:10.3945/ajcn.112.044545.

Supplementary Table S5. The estimated difference in ln-transformed homocysteine levels associated with GRS.

	Tertiles of genetic risk score			p-trend	Per increment of five risk alleles	
	T1	T2	T3		β (95% CI)	p
GRS[a]	<14.49	14.49-16.81	≥16.82			
Sample size	5040	5043	5043		15126	
β (95%CI)	0.000 (ref)	0.067 (0.051, 0.084)	0.191 (0.175, 0.208)	<0.001	0.162 (0.149, 0.175)	<0.001

CI = confidence interval; GRS = genetic risk score; ref = reference; SNP = single nucleotide polymorphism; T = tertiles.

Model adjusted for age and sex.

[a] Individual SNPs were coded as 0, 1, and 2 according to the number of risk alleles. The GRS = (β1×SNP1+β2×SNP2+…+β18×SNP18)×18/sum of the β coefficients, with the β coefficient for each SNP derived from the reported meta-analysis.

Supplementary Table S6. The estimated difference in ln-transformed homocysteine levels associated with GRS-6.

	Tertiles of genetic risk score			p-trend	Per increment of five risk alleles	
	T1	T2	T3		β (95% CI)	p
GRS-6[a]	< 5.31	5.30-6.91	≥ 6.92			
Sample size, n	4989	5082	5055		15126	
β (95%CI)	0.000 (ref)	0.051 (0.035, 0.068)	0.197 (0.181, 0.214)	<0.001	0.218 (0.202, 0.235)	<0.001

CI = confidence interval; GRS = genetic risk score; ref = reference; SNP = single nucleotide polymorphism; T = tertiles.

Model adjusted for age and sex.

[a] The GRS-6 = (β1×SNP1+β2×SNP2+…+β6×SNP6)×6/sum of the β coefficients, with the β coefficient for each SNP obtained from the present study.

Supplementary Figures

Supplementary Figure S1. Association of sleep duration and midday napping with serum homocysteine stratified by baseline characteristics. Generalized linear regression models were used, with (**A**) moderate sleep duration (7 to <8 hours) and (**B**) midday napping (1-30 minutes) as the reference groups, and adjusted for age (continuous), sex (female, male), education level (primary school or below, middle school, high school or higher), body mass index (continuous), presence of hypertension, dyslipidemia, and diabetes (yes, no), smoking status (current, former, never), drinking status (current, former, never), dietary intakes of meats, milk or dairy products, beans or soy products, fishes or seafoods, and fruits or vegetables (≥5 times/week; yes, no), regular exercise (yes, no), snoring (yes, no), sleep quality (good, fair, poor), midday napping (continuous), and sleep duration (continuous). Each group adjusted for the other covariates except itself. *P* for interactions were calculated by including the product terms of the stratified variables with sleep duration or midday napping in the multivariable-adjusted models, separately. BMI, body mass index; eGFR, estimated glomerular filtration rate; Hcy, homocysteine; CI, confidence interval.

Supplementary Figure S2. Serum homocysteine (Hcy) levels according to combined categories of sleep duration and midday napping. Means (standard error) were calculated with adjustments for age (continuous), sex (female, male), education level (primary school or below, middle school, high school or higher), body mass index (continuous), presence of hypertension, dyslipidemia, and diabetes (yes, no), smoking status (current, former, never), drinking status (current, former, never), dietary intakes of meats, milk or dairy products, beans or soy products, fishes or seafoods, and fruits or vegetables (≥5 times/week; yes, no), regular exercise (yes, no), snoring (yes, no) and

sleep quality (good, fair, poor). Hcy, homocysteine.

Supplementary Figure S3. Serum homocysteine (Hcy) levels according to combined categories of GRS-6 with sleep duration and midday napping. Bars are means (standard error) after adjustments for age (continuous), sex (female, male), education level (primary school or below, middle school, high school or higher), body mass index (continuous), presence of hypertension, dyslipidemia, and diabetes (yes, no), smoking status (current, former, never), drinking status (current, former, never), dietary intakes of meats, milk or dairy products, beans or soy products, fishes or seafoods, and fruits or vegetables (≥5 times/week; yes, no), regular exercise (yes, no), snoring (yes, no) and sleep quality (good, fair, poor); in addition, sleep duration (continuous) was adjusted for investigation of the interaction between GRS-6 and midday napping on Hcy, and midday napping (continuous) was adjusted for investigation of the interaction between GRS-6 and sleep duration on Hcy. The GRS-6 (lowest, median, or highest group) groups were defined by tertiles of the total population. *P* for interactions were calculated by including the product term of GRS-6 with sleep duration or midday napping in the multivariable-adjusted model, separately. GRS, genetic risk score; Hcy, homocysteine.

Supplementary Figure S4. The schematic overview of two major pathways of homocysteine metabolism. B6/B12, vitamin B6/B12; CBS, cystathionine β-synthase; CSE, cystathionine β-synthase; DPEP1, dipeptidase 1; GSH, glutathione; Hcy, homocysteine; MS, methionine synthase; MTHFR, methylenetetrahydrofolate reductase; SAH, S-adenosylhomocysteine; SAM, S-adenosylmethionine; THF, tetrahydrofolate.

A. Sleep duration ≥9 hours

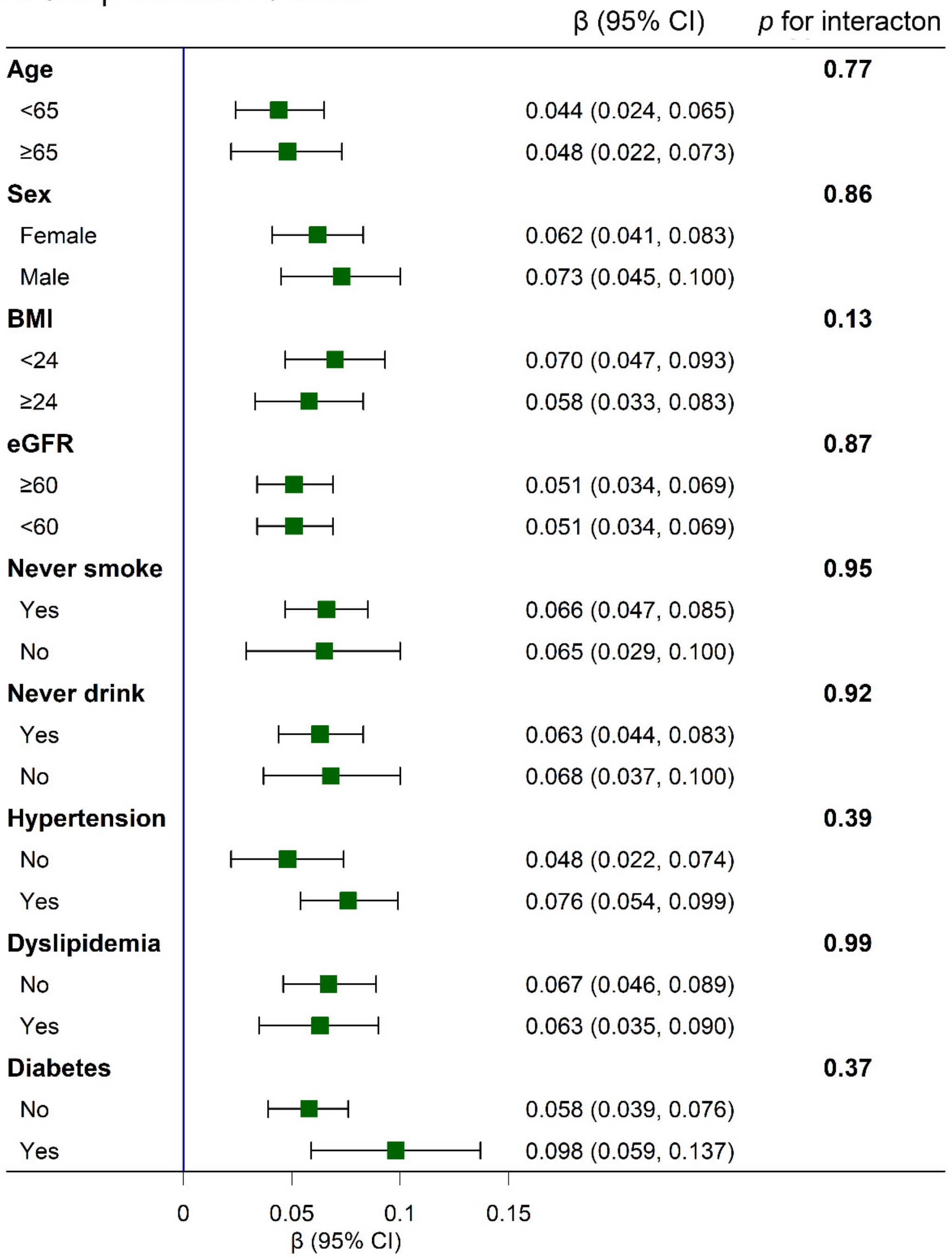

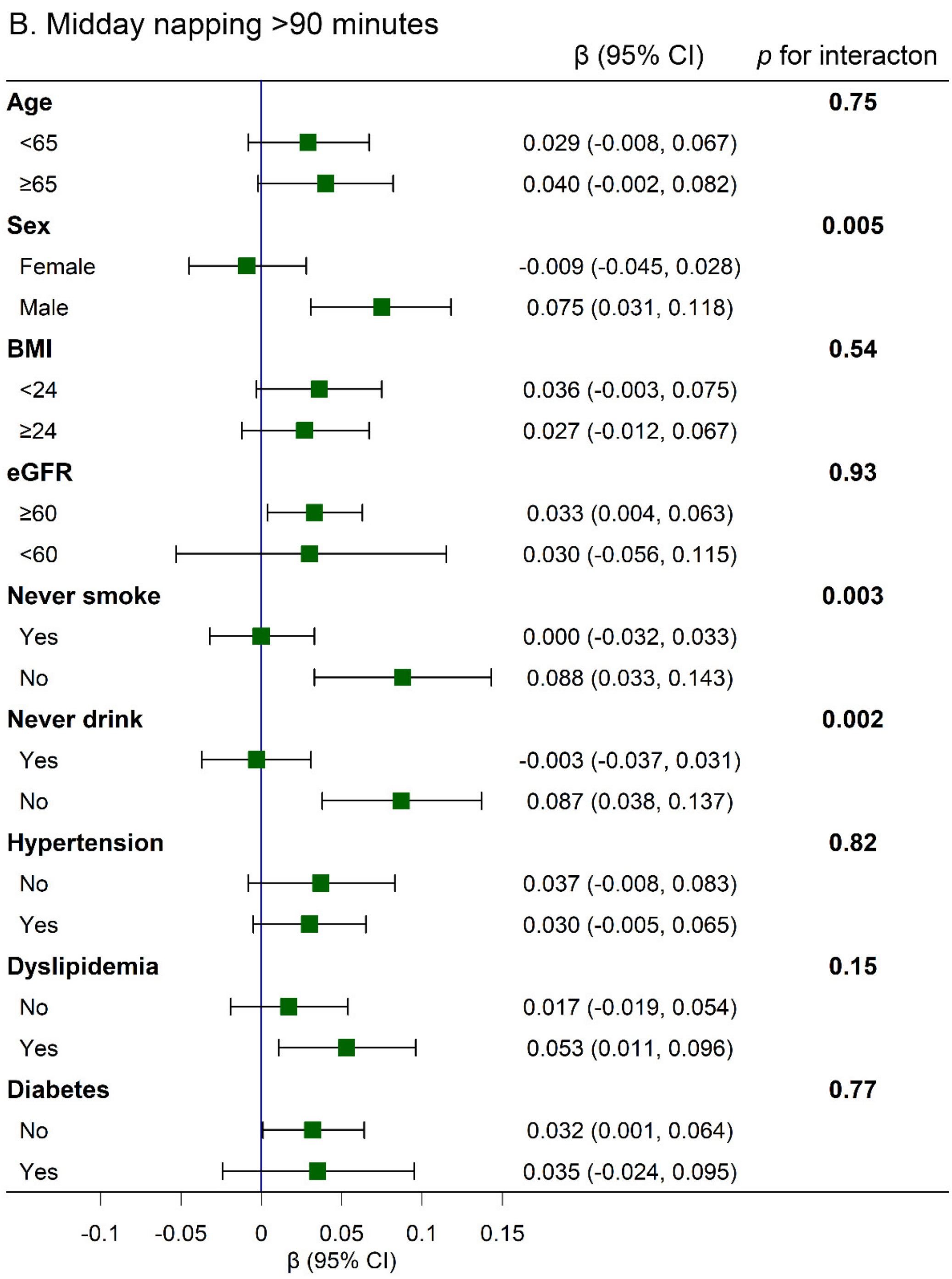

Supplementary Figure S1. Association of sleep duration and midday napping with serum homocysteine levels stratified by baseline characteristics.

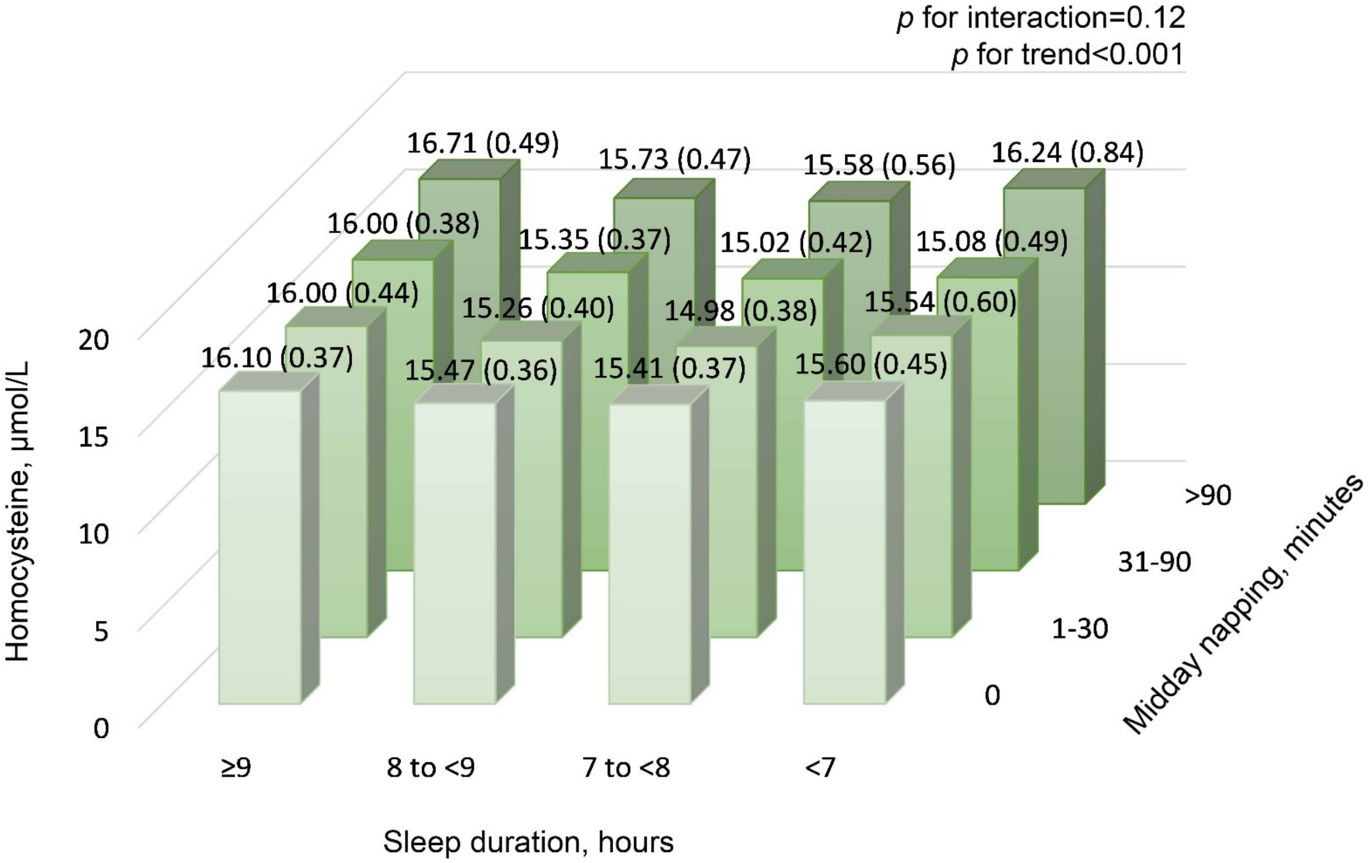

No.

≥9	8 to <9	7 to <8	<7	
391	440	236	79	>90
1753	2803	762	365	31-90
628	1215	1629	192	1-30
2558	3811	2068	496	0

Supplementary Figure S2. Serum homocysteine (Hcy) levels according to combined categories of sleep duration and midday napping.

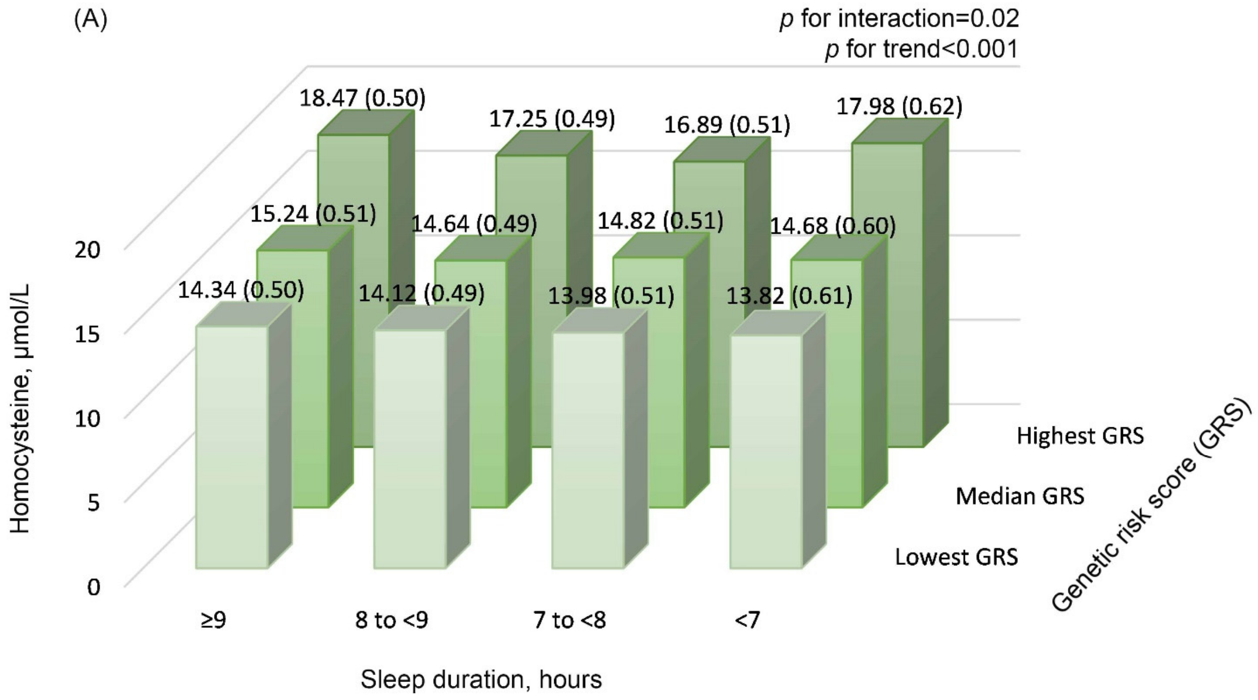

No.

	≥9	8 to <9	7 to <8	<7	
	1414	2170	1214	257	High GRS
	1363	2115	1270	334	Median GRS
	1350	2138	1207	294	Low GRS

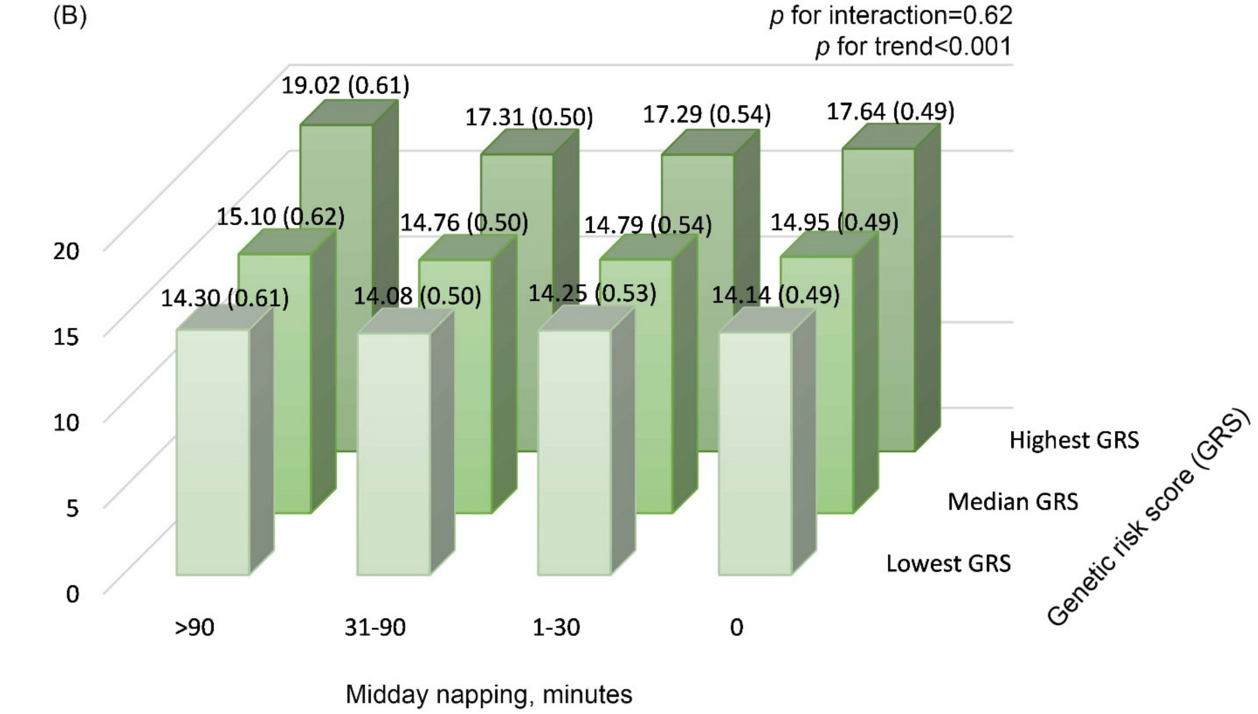

No.

	>90	31-90	1-30	0	
	303	1706	703	2343	High GRS
	282	1716	725	2359	Median GRS
	298	1705	760	2226	Low GRS

Supplementary Figure S3. Serum homocysteine (Hcy) levels according to combined categories of GRS-6 with sleep duration and midday napping.

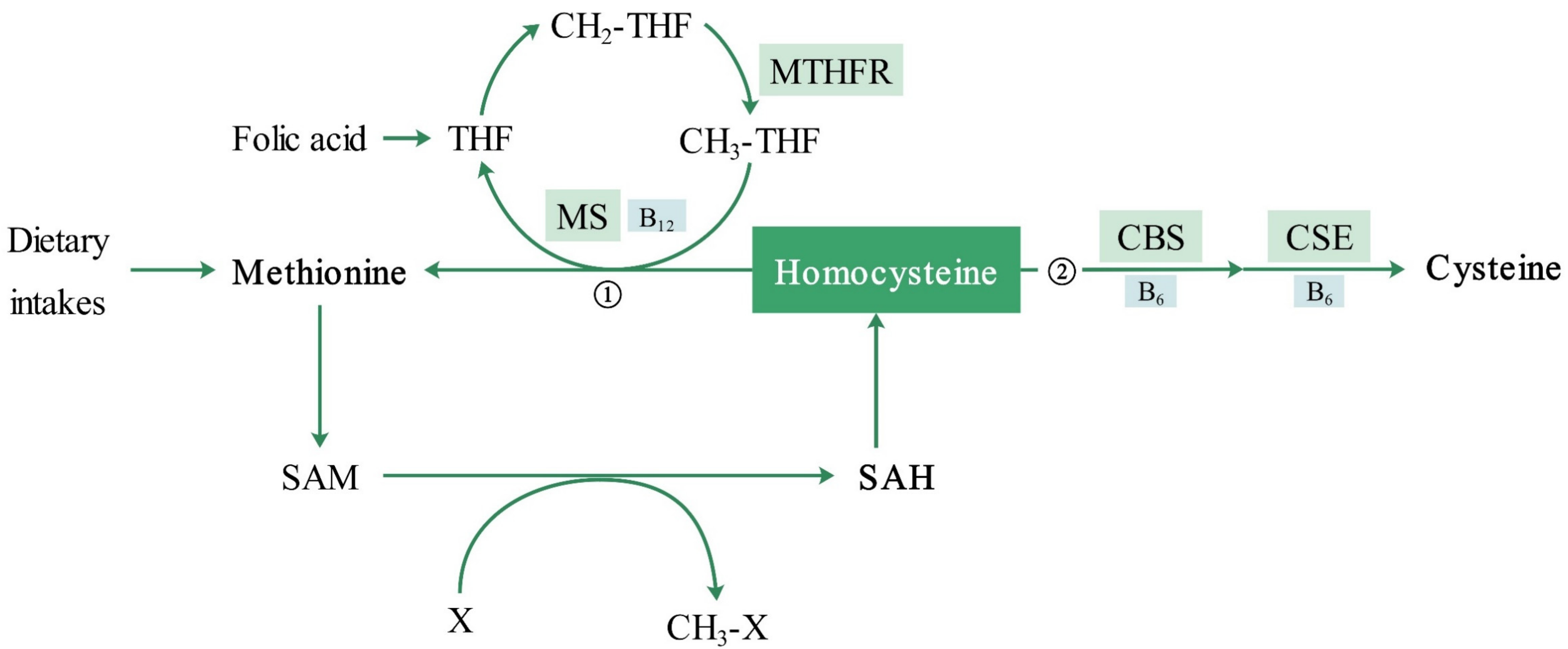

Supplementary Figure S4. The schematic overview of two major pathways of homocysteine metabolism.

Reference

1. Williams, C. Work-life balance of shift workers. *Perspectives on Labour and Income* **2008**, *9*.
2. Li, W.; Yu, K.; Jia, N.; Xu, X.; Yuan, Y.; Peng, R.; Niu, R.; You, X.; Yang, H.; Qiu, G., et al. Past Shift Work and Incident Coronary Heart Disease in Retired Workers: A Prospective Cohort Study. *American journal of epidemiology* **2021**, *190*, 1821-1829, doi:10.1093/aje/kwab074.
